# Nontuberculous mycobacterial disease following hot tub exposure.

**DOI:** 10.3201/eid0706.010623

**Published:** 2001

**Authors:** E J Mangione, G Huitt, D Lenaway, J Beebe, A Bailey, M Figoski, M P Rau, K D Albrecht, M A Yakrus

**Affiliations:** Colorado Department of Public Health & Environment, Disease Control & Environmental Epidemiology Division, Denver, 80246-1530, USA. ellen.mangione@state.co.us

## Abstract

Nontuberculous mycobacteria (NTM) have been recognized as an important cause of disease in immunocompromised hosts. Pulmonary disease caused by NTM is increasingly recognized in previously healthy persons. Investigation of pulmonary disease affecting a family of five identified an indoor hot tub as the source of NTM-related disease.


### Nontuberculous Mycobacterial Disease Following Hot Tub Exposure

**Ellen J. Mangione,* Gwen Huitt,† Dennis Lenaway,‡ James Beebe,* Ann Bailey,‡ Mary Figoski,§ Michael P. Rau,* Kurt D. Albrecht,* and Mitchell A. Yakrus¶**
*Colorado Department of Public Health & Environment, Denver, Colorado, USA; †University of Colorado Health Sciences Center, Denver, Colorado, USA; ‡Boulder County Health Department, Boulder, Colorado, USA; §University Family Medicine, Boulder, Colorado, USA; and Centers for Disease Control and Prevention, Atlanta, GA, USA

Nontuberculous mycobacteria (NTM) have been recognized as an important cause of disease in immunocompromised hosts. Pulmonary disease caused by NTM is increasingly recognized in previously healthy persons. Investigation of pulmonary disease affecting a family of five identified an indoor hot tub as the source of NTM-related disease.

Nontuberculous mycobacteria (NTM) are an important cause of disease in the United States, with the number of NTM isolates exceeding those of *Mycobacterium tuberculosis* (1). Pulmonary disease, the most commonly reported localized manifestation of NTM, is often associated with the *M.avium* complex (MAC) (2). Other NTM species, such as *M. kansasii, M. fortuitum, M. xenopi,* and *M. abscessus,* have also been associated with pulmonary disease (2,3). Although NTM-associated pulmonary disease has been described primarily among immunocompromised persons (4,5), it is being recognized with increasing frequency among those without predisposing conditions (2,6,7).

Unlike MTB, NTM are not known to be transmitted person to person. Most NTM have been isolated from water or soil (8-14). Species such as MAC are thermophilic (12), resistant to chemical germicides (14), and readily aerosolized (13). For several NTM species, environmental sources have been linked epidemiologically to cases of disease (15-20). In 1991, Burns investigated an outbreak of respiratory tract colonization in which epidemiologic and pulsed-field gel electrophoresis (PFGE) findings implicated a contaminated showerhead as the source of *M. fortuitum* (21). Subsequently, von Reyn used PFGE to link MAC infection in five AIDS patients to hot water sources in two hospitals (22).

Recently, Embil et al. (23) described five persons who became ill with pulmonary disease following exposure to hot tubs. MAC was isolated from all five patients and the two tubs. When MAC isolates were examined by multilocus enzyme electrophoresis (MEE), however, the hot tub and patient isolates had different MEE patterns. Kahana et al. (24) reported one patient diagnosed with MAC disease associated with a hot tub. In this case, the organisms isolated from the patient and the tub were identical by MEE.

Address for correspondence: Ellen J. Mangione, Colorado Department of Public Health & Environment, Disease Control & Environmental Epidemiology Division, 4300 Cherry Creek Drive South, Denver, CO 80246-1530, USA; fax: 303-758-9051; e-mail: ellen.mangione@state.co.us

### The Study

In October 1998, the Boulder County Health Department and the Tuberculosis Program, Colorado Department of Public Health and Environment, began an investigation into an apparent cluster of tuberculosis cases among a previously healthy family of five.

Case 1: This patient, a 46-year-old woman, was in excellent health until early June, when she noted the onset of shortness of breath and a dry cough. A chest radiograph at that time was consistent with early right lower lobe bronchial pneumonia. The patient was treated over the subsequent weeks with a series of antibiotics, including amoxacillin and azithromycin, without improvement. During early July, she had fever as high as 104°F approximately every 4 days, accompanied by night sweats and preceded by chills. A chest radiograph showed increased markings in the right lower lobe medially with associated peribronchial thickening and some faint air bronchograms consistent with an early bronchial pneumonia. She was begun on a course of tetracycline and prednisone (50 mg four times a day for 3 days, decreased to 20 mg four times a day for 11 days). During this time the patient traveled to Disneyland and while away felt much improved. Her symptoms recurred with the cessation of steroids and return home, however, and by the end of August, her shortness of breath, night sweats, and malaise had worsened, and she had an 18-pound weight loss.

Before she became ill, the patient had exercised regularly. In late August, her shortness of breath worsened so that she was unable to walk across a room, and she visited the hospital. A chest radiograph showed increased interstitial markings in both lungs. A computed tomographic (CT) scan of the chest showed a diffuse increase in pulmonary interstitial markings, with a ground-glass background. On September 10 the patient underwent fiberoptic bronchoscopy. A right lower lobe biopsy showed an occasional noncaseating granuloma consistent with sarcoidosis. Stains for acid-fast bacteria (AFB) and fungi were negative.

A chest radiograph obtained September 10 following bronchoscopy showed a fine reticular interstitial pattern involving the mid to lower lung. The patient was begun on a second course of prednisone and showed some improvement. A chest radiograph obtained on September 22, however, showed an interstitial lung process involving all areas, although both lungs appeared radiographically improved. Prednisone was discontinued on September 23 in anticipation of an open lung biopsy. Oxygen saturation ($SaO_2$) measurements before surgery on 4 liters of $0_2$ ranged from 88% to 92%.

On September 24, the patient had an open lung biopsy of the lingula and left lower lobe. The lingula showed moderate to severe granulomatous inflammation with AFB, numerous granulomas with focal caseation and necrosis, interstitial chronic inflammation, and mild and interstitial immature fibrosis with focal pattern. The left lower lobe of the lung showed moderate granulomatous inflammation, multiple granulomas with focal caseation and necrosis, and mild interstitial chronic inflammation and immature fibrosis. Aerobic tissue cultures showed rare gram-positive cocci. Stains and cultures for fungi and *Legionella* were negative, as were a shell vial assay and culture for cytomegalovirus. An intradermal test with 5 tuberculin units of purified protein derivative S (PPD) placed on September 24 was negative. Serologic HIV test results were also negative. A chest radiograph obtained September 25 showed bilateral bibasilar consolidation consistent with atelectasis or pneumonia with some infiltrate in the upper lobes.

On September 25, the patient was begun on oral isoniazid, 300 mg; rifampin, 600 mg; ethambutol, 400 mg; and pyrazinamide, 500 mg daily for suspected miliary tuberculosis. This regimen was discontinued 7 days later.

On subsequent evaluation at National Jewish Hospital in early October, the patient had $pO_2$ 40, $pCO_2$ 36, pH 7.43, and $SaO_2$ 77% on room air. High-resolution CT scan showed fine central lobular nodularity without bronchiectasis. Pulmonary function tests were remarkable (Table).

Case 2: The 44-year-old husband of Patient 1 was well until mid-August when he had onset of productive cough, fever, and night sweats. Over the next month, he had an 8-pound weight loss. His past medical history is remarkable only for a history of heavy smoking (1/2 to 1 pack per day) until 7 years previously, when he stopped smoking entirely.

Cases 3, 4, and 5: The 14-, 12-, and 9-year-old sons of Patients 1 and 2 became ill in mid-September with influenza-like symptoms, including fever as high as 104°F, nausea, vomiting, and shortness of breath. The 12-year-old (Patient 4) was hospitalized for dehydration. His chest radiograph showed diffusely abnormal lungs with mildly increased nodular markings more prominent in the lower than upper lobe. Pulmonary function tests were performed for Patient 4 in early October (Table).

A chest radiograph for Patient 3 showed ground-glass alveolar infiltration with tiny opacities, suggesting a miliary pattern consistent with tuberculosis. Patient 5's chest radiograph showed mild increased streaking bilaterally.

All family members had negative skin tests with PPD at 48 and 72 hours. Although none described potential exposures to tuberculosis, the prospect of a family with tuberculosis prompted retrieval of specimens and pending cultures from the local hospital. Probe tests at the state public health laboratory on these specimens and sediments of centrifuged culture medium were all negative for *M. tuberculosis* but positive for MAC for two family members. Because of these laboratory findings and the unusual occurrence of five cases of suspected pulmonary tuberculosis among family members, the cases were presented to a physician at National Jewish Hospital, who noted the possibility of NTM-related disease secondary to hot tub exposure.

### Environmental Investigation

Approximately a year earlier, the family had installed a hot tub in an enclosed sunroom next to the kitchen. The source of water for the tub was surface water from the Boulder municipal system, transported via tanker truck. Drinking and bathing water come from an alluvial aquifer well shared with several neighbors.

The hot tub water was changed only two or three times from January to October. The tub was equipped with an ozonator. On occasion a chlorine/bromine float or a cup of bleach would be added just before the tub was used. Disinfectant levels or pH were not checked.

Patient 1 used the hot tub rarely, most recently once in June and a second time in July. Because the tub water irritated her skin, she showered immediately after using the tub. However, when one of her sons was in the tub, she generally stood nearby. The 12-year-old, Patient 4, was the most frequent user. The three children often entered the tub after having been outside, without having showered first. Patients

Table. Laboratory and pulmonary function test results for five patients with nontuberculous *Mycobacterium* infection

| | Sputum tests | | Pulmonary function tests | | | | | | | |
|---|---|---|---|---|---|---|---|---|---|---|
| | | | $FEV_1$ | | FVC | | DLCO/VA | | RV | |
| Patient | AFB[a] | MAC | initial | 6 mo | initial | 6 mo | initial | 6 mo | initial | 6 mo |
| 1 | - smear | + culture | 1.40 (42%) | 2.47 (75%) | 1.86 (44%) | 3.25 (80%) | 82% | 87% | 179% | 147% |
| 2 | + smear | + culture | -- | | -- | | -- | | -- | |
| 3 | - smear | + culture | -- | | -- | | -- | | -- | |
| 4 | + smear + culture | - probe | 1.98 (61%) | 2.63 (76%) | 2.57 (68%) | 3.41 (84%) | 91% | 98% | 226% | 88% |
| 5 | - smear - culture | -- | -- | | -- | | -- | | -- | |

[a]AFB = acid-fast bacilli; MAC = *Mycobacterium avium* complex; $FEV_1$ = forced expiratory volume; FVC = forced vital capacity; DLCO/VA = diffusing capacity of the lung for carbon monoxide; RV = residual volume; – = not done.

1 and 4 had the greatest exposure to the hot tub aerosols. In retrospect, they described a clear relationship between hot tub exposure and worsening of symptoms, i.e., recurrence of night sweats, chills, and fever.

Following identification of MAC and *M. fortuitum* from clinical specimens (Table) and further consultation, Patients 1 and 4 were begun on a regimen of rifampin, ethambutol, amikacin, clarithromycin, ciprofloxacin, and prednisone. Patients 2, 3, and 5 were treated with clarithromycin and ciprofloxacin. After 6 months, pulmonary function tests for Patients 1 and 4 had improved (Table), signs and symptoms had resolved, and chest radiographs were normal for all family members.

The results of sputum evaluation on smear and culture for AFB are summarized (Table). Patient 4 was smear and culture positive for AFB, but negative on probe for MTB or MAC. The organism was subsequently identified as *M.foruitum*.

Patient and water isolates were initially identified as MAC and typed by MEE, with identical enzyme profiles (25). Restriction fragment-length polymorphism (RFLP) analysis with an insertion sequence specific for *M. avium* (IS *1245*) (26) confirmed that all isolates were the identical strain of *M. avium* (Figure). Isolation of *M. fortuitum* from this hot tub has been described (27).

### Conclusions

NTM organisms isolated from the hot tub are likely responsible for this family's illness for the following reasons: exposure to the hot tub was temporally related to onset of symptoms; MAC was isolated from the lung biopsy and sputum of one patient and the sputum of two others, as well as from the hot tub; and MAC isolates from patient specimens and the hot tub were identical by RFLP. In addition, *M. fortuitum* was isolated from both the hot tub and a fourth hot tub-exposed person.

The source of the MAC and *M. fortuitum* is unclear. Our inability to isolate either organism from samples from the tanker truck used to supply water for the hot tub does not rule this out as a source. NTM have been isolated from municipal water supplies in the past (22). Alternatively, the users may have introduced the organisms, as the children often used the tub without showering first.

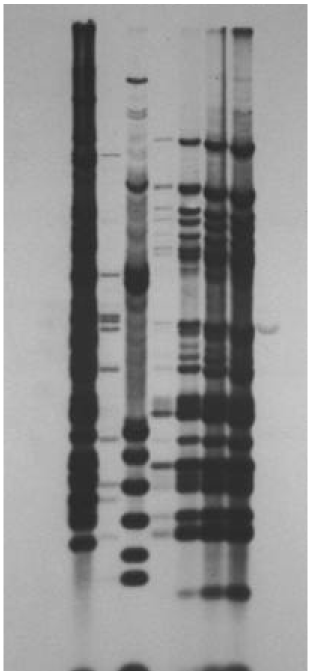

Figure. Restriction fragment-length polymorphism analysis for isolates from patients and hot tub. Lane 1: *Mycobacterium avium* CDC #91-9282, serotype 4. Lane 2: *M. avium* CDC #91-9285, serotype 10. Lane 3: *M. avium* CDC #91-9299, serotype 8. Lane 4: Hot tub isolate. Lane 5: Isolate from Patient 2. Lane 6: Isolate from Patient 3. Lane 7: Isolate from Patient 1.

Proliferation of these organisms in a hot tub is not surprising, as both MAC and *M. fortuitum* are thermophilic (12). Moreover, at temperatures >84°F, chlorine loses much of its efficacy as a disinfectant (15).

Controversy exists as to whether persons with pulmonary disease secondary to NTM are experiencing a hypersensitivity reaction to the organisms or symptoms secondary to true infection (28,29). Murphy concludes that in the presence of dyspnea, nodular infiltrates seen on CT, response to steroids, and absence of predisposing factors such as chronic lung disease, a patient with MAC-related lung disease has hypersensitivity pneumonitis. Pathologic findings, including palisaded and multinucleated histiocytes and granulomatous inflammation, however, suggest infection (28). In a recent case presentation, symptoms and radiographic findings in a patient from whose lung tissue MAC was cultured are consistent with both the diagnoses of hypersensitivity pneumonitis and atypical mycobacterial infection, a conclusion substantiated by pathologic findings (29). In cases evaluated at National Jewish Medical and Research Center, most patients required treatment with both steroids and antimycobacterial medications (30). This experience suggests that NTM disease represents a spectrum of disease with components of both hypersensitivity pneumonitis and infection.

Our cases had characteristics of both hypersensitivity pneumonitis and true infection. The short interval between hot tub use and exacerbation of symptoms and the patchy ground-glass appearance of the lungs, with centrilobular nodules on CT, suggest hypersensitivity pneumonitis (31). The granulomas seen on pathologic examination and the response to treatment with antimycobacterial medications, however, suggest true infection. The temporary improvement in Patient 1's condition after she received prednisone may represent either appropriate treatment of hypersensitivity pneumonitis or a decrease in granulomatous inflammation in the bronchioles, secondary to infection (28).

Little data exist to explain the mechanism of disease caused by NTM in healthy persons. Exposure to sufficiently large and repeated inocula of the organism in droplets of readily respirable size appears to be sufficient to overwhelm normal host defenses.

Hot tubs should be maintained according to manufacturers' recommendations, which include both frequent water changes and adequate use of disinfectants. In addition, placing a hot tub in an enclosed environment should be strongly discouraged. Patients with atypical pneumonia should be questioned about similar illnesses among family member and others who have had similar exposures, including exposure to a hot tub. As hot tubs become increasingly popular (pers. comm., John J. Cergol, Jr.), hot tub-related illness associated with NTM may become an emerging infectious disease challenge.

### Acknowledgment

We thank Sally G. Houser for preparation of this manuscript.

Dr. Mangione is director of the Disease Control and Environmental Epidemiology Division of the Colorado Department of Public Health and Environment. She holds appointments in the Department of Biometrics and Preventive Medicine and the Division of Infectious Diseases, University of Colorado Health Sciences Center.

### References

1. Ostroff S, Hutwagner L, Collin S. Mycobacterial species and drug resistance patterns reported by state laboratories–-1992. 93rd American Society for Microbiology General Meetings; 1993 May 16; Atlanta, GA. Abstract U-9. p. 170.
2. O'Brien RH, Geiter LJ, Snider DE. The epidemiology of nontuberculous mycobacterial diseases in the United States. Am Rev Respir Dis 1987;135:1007-14.
3. Griffith DE, Girard WM, Wallace RJ. Clinical features of pulmonary disease caused by rapidly growing mycobacteria: An analysis of 154 patients. Am Rev Respir Dis 1993;147:1271-8.
4. Rosenzweig DY. Pulmonary mycobacterial infections due to *Mycobacterium intracellulare-avium* complex: clinical features and course in 100 consecutive cases. Chest 1979;75:115-9.
5. Horsburgh CR. *Mycobacterium avium* complex infection in the acquired immunodeficiency syndrome. N Engl J Med 1991;324:1332-8.
6. Huang JH, Kao PN, Adi V, Ruoss SJ. *Mycobacterium avium-intracellulare* pulmonary infections in HIV negative patients without pre-existing lung disease. Chest 1999;115:1033-40.
7. Prince DS, Peterson DD, Steiner RM, Gottlieb JE, Scott R, Israel HL, et al. Infection with *Mycobacterium avium* complex in patients without predisposing conditions. N Engl J Med 1989;321:863-8.
8. Collins CH, Grange JM, Yates MD. Mycobacteria in water. J Appl Bacteriol 1984;57:193-211.
9. Wolinsky E, Rynearson TK. Mycobacteria in soil and their relation to disease-associated strains. Am Rev Respir Dis 1968;97:1032-7.
10. Reznikov M, Leggo JH, Dawson DJ. Investigation by seroagglutination of strains of *Mycobacterium intracellulare-M. scrofulaceum* group from house dusts to sputum in southeastern Queensland. Am Rev Respir Dis 1971;104:951-3.
11. Gruft H, Falkinham JO, Parker BC. Recent experience in epidemiology of disease by atypical mycobacteria. Rev Infect Dis 1981;3:990-6.
12. duMoulin GC, Stottmeier KD, Pelletier PA, Tsang AY, Hedley-Whyte J. concentration of *Mycobacterium avium* by hospital hot water systems. JAMA 1988;260:1599-601.
13. Parker BC, Ford MA, Gruft H, Falkinham JO. Epidemiology of Infection by nontuberculous mycobacteria: IV. Preferential Aerosolization of *Mycobacterium intracellulare* from natural waters. Am Rev Respir Dis 1983;128:652-6.
14. Wendt SL, George KL, Parker BC, Gruft H, Falkinham JO. Epidemiology of infection of nontuberculous mycobacteria: III. Isolation of potentially pathogenic mycobacteria from aerosols. Am Rev Respir Dis 1980;122:259-63.
15. Pelletier PA, duMoulin GC, Stottmeier KD. Mycobacteria in public water supplies: Comparative resistance to chlorine. Microbiol Sci 1988;5:147-8.
16. Costrini AM, Maher DA, Gross WM, Hawkins JE, Yesner R, D'Esopo M. Clinical and roentgenographic features of nosocomial pulmonary disease due to *Mycobacterium xenopi*. Am Rev Respir Dis 1981;123:104-9.
17. Bolan GA, Reingold AL, Carson LA, Silcox VA, Woodley CL, Hayes PS. Infections with *Mycobacterium chelonei* in patients receiving dialysis and using processed dialyzers. J Infect Dis 1985;152:1013-9.
18. Lowry PW, Jarvis WR, Oberle AD, Bland LA, Silberman R, Bocchini JA. *Mycobacterium chelonae* causing otitis media in an ear-nose-and throat practice. N Engl J Med 1988;319:978-82.
19. Lockwood WW, Friedman C, Bus N, Pierson C, Gaynes R. An outbreak of *Mycobacterium terrae* in clinical specimens associated with a hospital potable water supply. Am Rev Respir Dis 1989;140:1614.
20. Yajko DM, Chin DP, Gonzalez PC, Nassos PS, Hopewill PC, Reingold AL, et al. *Mycobacterium avium* complex in water, food and soil samples collected from the environment of HIV–infected individuals. J Acquir Immune Defic Syndr Hum Retrovirol 1995;9:176-82.
21. Burns DN, Wallace RJ, Schultz ME, Zhang Y, Zubairi SQ, Pang Y, et al. Nosocomial outbreak of respiratory tract colonization with *Mycobacterium fortuitum*: Demonstration of usefulness of pulsed-field gel electrophoresis in an epidemiologic investigation. Am Rev Respir Dis 1991;144:1153-9.
22. VonReyn CF, Maslow JN, Barber TW, Falkinham JO, Arbeit RD. Persistent colonisation of potable water as a source of *Mycobacterium avium* infection in AIDS. Lancet 1994;343:1137-41.
23. Embil J, Warren P, Yakrus M, Stark R, Corne S, Forrest D, et al. Pulmonary illness associated with exposure to *Mycobacterium-avium* complex in hot tub water: Hypersensitivity pneumonitis or infection? Chest 1997;111:813-16.
24. Kahana LM, Kay JM, Yakrus MA, Waserman S. *Mycobacterium avium* complex infection in an immunocompetent young adult related to hot tub exposure. Chest 1997;111:242-5.
25. Yakrus MA, Reeves MW, Hunter SB. Characterization of isolates of *Mycobacterium avium* serotypes 4 and 8 from patients with AIDS by multilocus enzyme electrophoresis. J Clin Microbiol 1992;30:1474-8.
26. Guerrero C, Bernasconi C, Burki D, Bodmer T, Telenti A. A novel insertion sequence from *Mycobacterium avium*, IS *1245*, is a specific target for analysis of strain relatedness. J Clin Microbiol 1995;33:304-7.
27. Martyny JW, Rose CS. Nontuberculous mycobacterial bioaerosols from indoor warm water sources cause granulomatous lung disease. Indoor Air 1999;9:1-6.
28. Murphy RLH, Mark EJ. Case 6-1996. a 40-year-old man with cough, increasing dyspnea, and bilateral nodular lung opacities. N Engl J Med 1996;334:521-6.
29. Schwartzstein RM, Mark EJ. Case 27-2000. A 61-year-old man with rapidly progressing dyspnea. N Engl J Med 2000;343:642-9.
30. Rose CS, Martyny J, Huitt G, Iseman M. Hot tub associated granulomatous lung disease from mycobacterial bioaerosols. Am J Respir Crit Care Med 2000;161:A730.
31. Lynch DA, Rose CS, Way D, King TE Jr. Hypersensitivity pneumonitis: Sensitivity of high-resolution CT in a population-based study. AJR Am J Roentgenol 1992;159:469-72.
